# School Health and Nutrition Monitoring: What Practitioners and Policy Makers Can Learn from China

**DOI:** 10.1016/j.lanwpc.2021.100368

**Published:** 2022-01-02

**Authors:** Linda Schultz, Donald A.P. Bundy

**Affiliations:** Research Consortium for School Health and Nutrition, London School of Hygiene and Tropical Medicine, London, UK

“Coverage of School Health Monitoring Systems in China: a Large National Cross-Sectional Survey” by Yan et al. provides an important demonstration of the value of monitoring national school health and nutrition programs.^1^ School-based surveys have long been used as cost-efficient monitoring tools, as they serve as a proxy for the broader health and wellbeing for the community in which they live and inform programmatic responses. Despite their importance, there is surprisingly little published on how countries operationally monitor their programs, which presents meaningful challenges for program implementers globally.^2^

Investing in the first 8,000 days of life – from conception until young adulthood – is necessary to ensure that children born today achieve their full potential by the time they enter the workforce. Investments in the first 1,000 days of life are critically important as they lay the foundation for good health and development. It is now increasingly recognized that those gains need to be secured and sustained with appropriate investments over the next 7,000 days, and complemented by quality interventions at critical developmental periods throughout middle childhood and adolescence. School health and nutrition programs are a cost-effective way to secure these objectives and ensure that learners are best positioned to take advantage of educational opportunities as they mature.^3^

School health and nutrition have increasingly become a priority for policy makers as a response to the COVID-19 pandemic, as these programs draw children to school and serve as a delivery platform for complementary health services. Economic evaluations have concluded that school-based health and nutrition interventions are a cost-effective contribution to the Universal Health Coverage package.^4^

China has an unusually complete school health and nutrition program, which is part of a larger investment across the 8,000 days. Since 2011, China has targeted health and education investments from birth to first job, especially for “Left Behind Children” (children who remain, usually in rural areas, while their parents seek opportunities in urban centers). The approach of this program, particularly for school-age children, was informed by the multi-sectoral approach described, for example, in the 2009 World Bank Rethinking School Feeding report.^5^ The effectiveness of this approach was demonstrated earlier this year when, after ten years of implementation, China declared an end of absolute poverty in the country.^6^

The 2018 Monitoring Questionnaire for School Teaching and Living Facilities Health Status surveys three aspects of school health and nutrition monitoring: infectious disease, non-communicable diseases, and school physical environments in China.

On infectious disease, we learn that nine-out-of-ten schools assessed have an established process for documenting vaccination status. This high compliance doubtless reflects the simplicity of this most logistically straightforward of the three monitoring aspects, and is also a reflection of the high priority that China has placed on vaccination management following the SARS outbreak in 2003. As other countries strengthen their national school health and nutrition programs in response to the COVID-19 pandemic, there may be important opportunities to learn from China's two decades of operational experience in this area.

On non-communicable disease, health status is monitored by annual medical examinations. The coverage of this aspect is much lower than for the monitoring of infectious disease, which doubtless reflects the much more complex procedures and management required to undertake physical/clinical examinations. The benefits of such a complex and expensive process are less clear, and have yet to be demonstrated for other countries. More research might be helpful to decide whether this strategy is cost-effective, and in particular whether it has a significant effect in reducing illness. Some aspects, in particular measuring height and weight, might be valuable to track overweight and obesity, especially around the pubertal growth spurt.

On school physical environment, it is compelling to learn that classroom ventilation is assessed, as this has become a central tactic for mitigating infection transmission in the COVID-19 pandemic. In addition, the authors note that classroom lighting is also now monitored due to concerns that it might contribute to the observed high rates of myopia. In a related move, China has adopted innovative ways to address the development of myopia, such as exploring the value of building breaks from close work into the school curriculum.

One important deficit in the current monitoring approach which is highlighted in this paper is the lack of focus on the nutritional composition and dietary diversity of the meals served. This is a missed opportunity as the value of school meals is dependent on their nutritional quality. We see an opportunity to refine the survey – not only to track the quality of school food served to 40 million children daily across China^7^ – but also to proactively address the increasing rates of overweight and obesity among its school-age children and adolescents.

The School Meals Coalition, launched at the Food Systems Summit in September 2021, is underpinned by the vision of ensuring the quality of school meals.^8^ To date, 61 countries have signed onto the Coalition to restore school-based nutrition services post pandemic. China has yet to decide whether to join this Coalition, but would have rich experience to share with other countries, especially around the processes for introducing, scaling, and strengthening its Nutrition Improvement Plan for Rural and Compulsory Education Students program.^9^

There is also an additional opportunity that China might wish to consider. To strengthen the evidence base and provide policy makers with mission-critical information, the Coalition membership has established a Research Consortium for School Health and Nutrition.^10^ The Research Consortium aims to improve the evidence to enhance the quality of national programmes for children and adolescents over the next 7,000 days of life. A case study on the China national program would make an important contribution to the current efforts to document how national school meal programs can be effectively designed, financed, delivered, and monitored.

## Declaration of Competing Interest

The authors declare no conflicts of interest.
